# How I do it: retrosigmoid intradural inframeatal petrosectomy

**DOI:** 10.1007/s00701-020-04587-0

**Published:** 2020-09-28

**Authors:** Torstein R. Meling, Greg Zegarek, Karl Schaller

**Affiliations:** 1 Division of Neurosurgery, Department of Clinical Neurosciences, Geneva University Hospitals, Geneva, Switzerland; 2 Faculty of Medicine, University of Geneva, Geneva, Switzerland; 3 Department of Neurological Surgery, Istituto Nazionale Neurologico “C.Besta”, Milan, Italy

**Keywords:** Surgery, Neurosurgery, Anatomy, Petrosectomy, Retrosigmoid, Brain tumor, Skull base

## Abstract

**Background:**

Lesions infiltrating the petrous temporal bone are some of the most complex to treat surgically. Many approaches have been developed in order to address these lesions, including endoscopic endonasal, anterior petrosectomy, posterior petrosectomy, and retrosigmoid.

**Method:**

We describe in a stepwise fashion the surgical steps of the retrosigmoid intradural inframeatal petrosectomy.

**Conclusion:**

The retrosigmoid intradural inframeatal petrosectomy may afford satisfactory exposure with limited drilling and minimal disruption of perilesional anatomical structures. It can provide excellent surgical results, especially for soft tumors, while minimizing surgical morbidity.

**Electronic supplementary material:**

The online version of this article (10.1007/s00701-020-04587-0) contains supplementary material, which is available to authorized users.

## Introduction

**Fig. 1:**
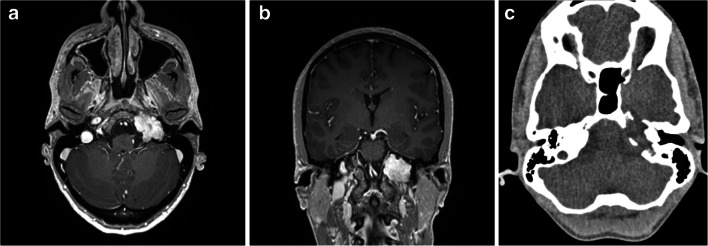
Preoperative T1 Gd+ MRI demonstrating the chondrosarcoma in the petrous apex left side on axial **a** and coronal **b** cuts. Preoperative CT demonstrating the chondrosarcoma in the petrous apex left side on axial **c** cuts

Lesions infiltrating the petrous temporal bone are certainly among the most difficult to treat in neurosurgery. Multiple approaches have been developed in order to access the petroclival space, and which approach is chosen should be assessed on a case-by-case basis [6, 8]. Each of these approaches, such as the endoscopic endonasal [2, 10], subtemporal [3], or anterior or combined petrous [5, 7] routes comes with certain built-in surgical comorbidities that must be accepted and explained to the patient [1]. We present a case of a grade II chondrosarcoma of the petrous bone (Fig. 1) operated (TRM) via the retrosigmoid intradural inframeatal petrosectomy (RESIP), and aim to discuss the advantages, but also the pitfalls and challenges involved.

## Relevant surgical anatomy

Multiple key anatomic landmarks must be evaluated [4, 9]. We rely on craniometric landmarks adjusted based on patient individual anatomy from preoperative imaging as well as neuronavigation and augmented reality. In planning the craniotomy, the transverse (TS) and sigmoid sinus (SS) must be identified. In charting the surgical trajectory, the pneumatization of the mastoid bone, the caudal cranial nerves (CNs), the jugular bulb, the superior and inferior petrosal sinuses (SPS, IPS), the acoustico-facial bundle, the internal acoustic canal (IAC), the petrous apex, the carotid canal, and Eustachian tube must be taken into account and outlined for the use of neuronavigation and augmented reality.

## Description of the technique

### Patient positioning and preparation

The patient is positioned in a supine position, with elevation of the ipsilateral shoulder. The head is fixed in a Doro® head-clamp and turned 100° contralaterally with the vertex turned slightly downwards. Brainlab® neuronavigation and augmented reality are installed, and the incision and craniotomy are planned. Markings are placed on the skin for the asterion, the trajectory of the TS and SS, and a curvilinear skin incision. Inomed® neuromonitoring is installed for auditory evoked potentials and caudal cranial nerves V-XII.

### Skin incision and soft tissue dissection

After minimal shaving and standard draping, a curvilinear, retroauricular skin incision is made extending from the mastoid tip, posterior to the asterion, and superior to the TS. Using a periosteal elevator, the skin and occipital muscles are elevated, and a self-retaining retractor is placed.

### Retrosigmoid craniotomy

The asterion is localized, and the TS and SS are verified with neuronavigation. A single burr hole is performed with a 50-mm cutting burr just medial and inferior to the TS-SS junction. A 2.5 × 3.0 cm craniotomy is performed. The SS and TS are further deskeletonized with the cutting burr. Mastoid air cells are opened in order to allow sufficient inferior access. The air cells are ablated with bone wax before the dural opening.

### Intradural dissection

**Fig. 2:**
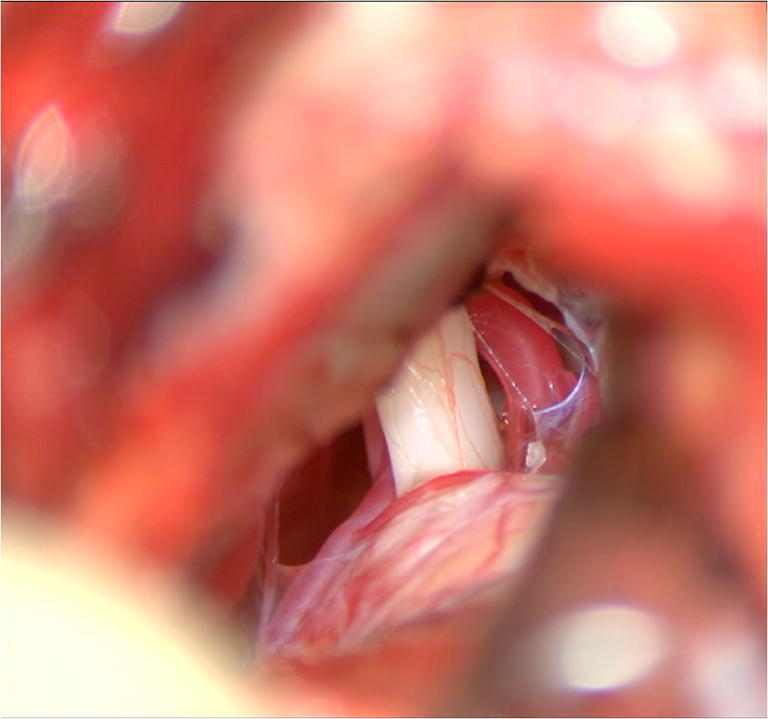
The dura has been incised and the CN VII/VIII bundle is identified as well as the AICA

The dura is opened following a C-shaped incision along the SS. The arachnoid membrane of the posterior wall of the cerebello-medullary cistern is opened. CSF is drained by aspiration until sufficient cerebellar relaxation is achieved. The jugular foramen, the hypoglossal canal, as well as the caudal cranial nerves and the CN VII/VIII bundle, are identified (Fig. 2).

### Inframeatal petrosectomy

**Fig. 3:**
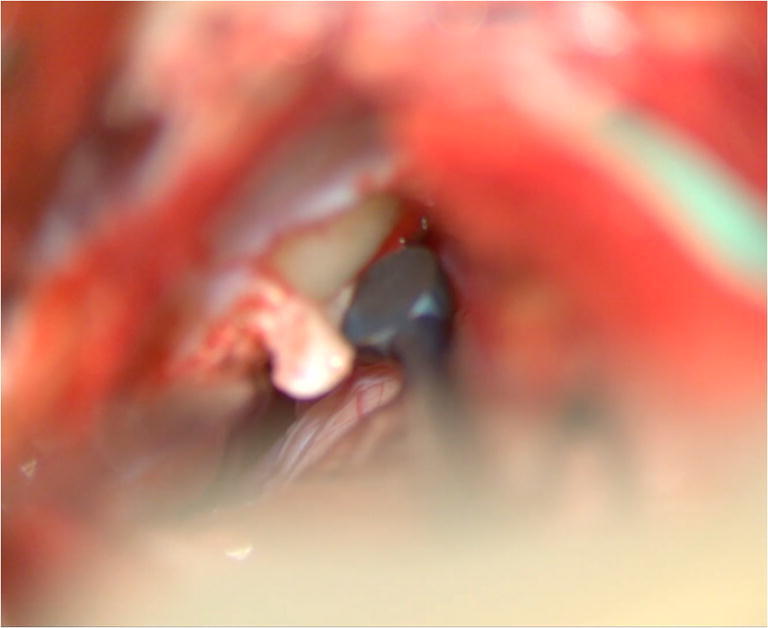
The dura overlying the inframeatal petrous bone is incised and dissected

**Fig. 4:**
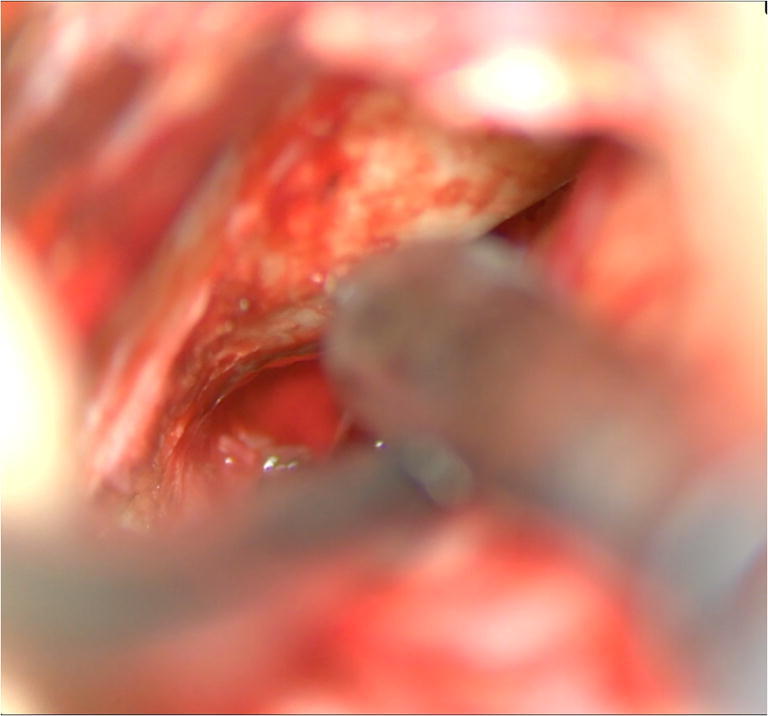
The inframeatal bone is drilled to expose the exophytic portion of the tumor. Drilling is continued towards the IAC until exposure of CN VII/VIII is achieved

The inframeatal dura is identified between the internal auditory meatus and the jugular bulb. The periosteum is incised with a knife and peeled off with a Rhoton dissector #3 (Fig. 3). A match stick 2-mm sharp burr (Medtronic® T9MH20 drill bit) is utilized to remove the bone overlying the tumor and then up to unfloor the IAC with visualization of the CN VII/VIII bundle (Fig. 4). The trajectory towards the petrous apex antero-medially is utilized.

### Tumor resection

The surgical corridor begins inferiorly to the CN VII/VIII bundle with a trajectory medial to the semicircular canals and the fallopian canal. In addition, the greater superficial, lesser petrosal, and deep petrosal nerves are protected. The resection terminates on reaching the carotid canal.

**Fig. 5:**
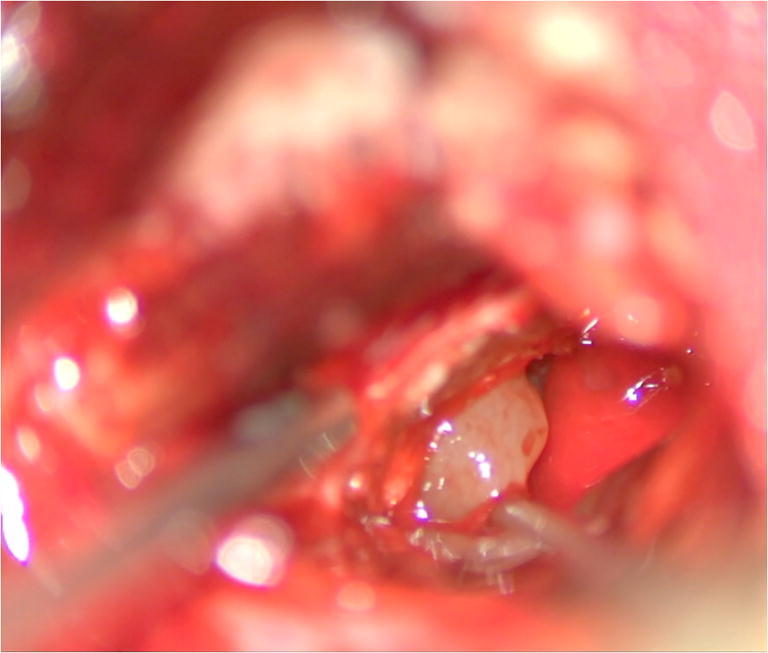
Tumor resection by angled curettes and aspiration is performed. The anterior limit of tumor resection is the petrous segment of the internal carotid artery

In this case, the tumor was quite soft, wherefore resection was performed by aspiration and angled curettes (Fig. 5). The cavity can be inspected and the resection can be completed under visual guidance using an endoscope.

Important venous bleeding might be encountered from the IPS during tumor resection which is controlled by Sutter® bipolar electrocautery or Gelfoam®, compression, and irrigation.

The inframeatal approach affords a sufficient surgical corridor from a simple retrosigmoid craniotomy while precipitating less surgical comorbidity compared with anterior petrosectomy [1, 10] or endoscopic endonasal approaches.

### Closure

The dural defect over the jugular foramen is closed with TachoSeal®. Water-tight dural closure is performed with Monocryl 5.0 running suture. Mastoid air cells are closed again with bone wax. The bone flap is repositioned using Stryker® titanium microplates and 4 mm self-tapping screws. Mastoid air cells are again closed with bone wax. Muscle and skin are closed with a running Monocryl 2.0 suture.

### Postoperative course

**Fig. 6:**
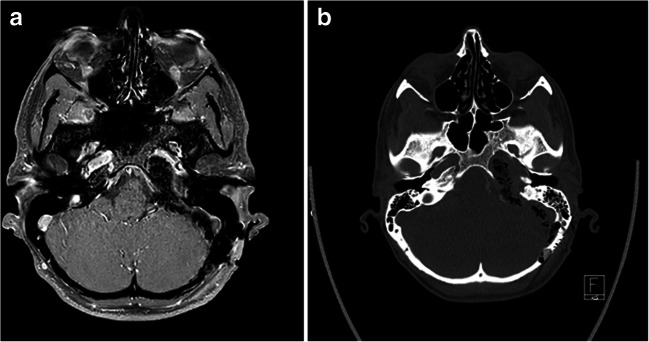
Postoperative imaging demonstrating near-total resection of the chondrosarcoma in the petrous apex left side on axial T1 Gd+ MRI subtraction series (**a**) and CT (**b**)

Postoperatively, our patient had an uneventful recovery with no CN deficits and no complications. She was mobilized the first postoperative day, and an MRI demonstrated a near-total resection of her chondrosarcoma (Fig. 6).

## Indications

Smaller petroclival tumors or larger soft or cystic petroclival lesions.

## Limitations

Upper petrous ridge tumors are limited by acoustic-facial bundle. Venous anatomy in case of high jugular bulb position precludes any drilling under and behind the acoustico-facial porus.

## How to avoid complications

- Careful preoperative anatomic study
- Augmented reality aid the surgical planning
- Meticulous microsurgical technique
- Cranial nerve neuromonitoring
- Careful closure to avoid CSF leak

## Specific perioperative considerations

### Preoperative workup

- Complete ENT workup
- MRI and high-definition CT of the skull base including angiography and venography
- Neuronavigation and augmented reality facilitates preoperative planning

### Postoperative care

- Bed rest overnight
- Head of bed at 30°
- Prophylactic anticoagulation to be started first postoperative day
- Systolic blood pressure below 120 mmHg
- Control MRI first postoperative day
- Multidisciplinary discussion at skull base tumor board

## Specific information to give to patients: surgery and potential risks

- Standard neurosurgical complications such as bleeding, infection, and CSF leak should be discussed.
- Given the particular anatomy, additional risks include damage to adjacent cranial nerves in the jugular foramen and the CN VII/VIII bundle, and the ICA and the vertebrobasilar arterial system, as well as risk of injury to the Eustachian tube. In specific, there is a risk of facial palsy, hearing loss, vertigo, dysphagia, cerebellar ataxia, stroke, and death.
- Depending on the pathology, complementary treatment may be proposed, such as proton therapy.

## Electronic supplementary material

ESM 1 Video demonstrating the retrosigmoid intradural inframeatal petrosectomy (RESIP) approach. (MOV 375186 kb).
